# Niveles de micronutrientes en niños escolares colombianos e inseguridad alimentaria

**DOI:** 10.7705/biomedica.5866

**Published:** 2021-09-22

**Authors:** Constanza Marín, Henry Oliveros, Eduardo Villamor, Mercedes Mora

**Affiliations:** 1 Facultad de Medicina, Universidad de La Sabana, Chía, Colombia Universidad de la Sabana Facultad de Medicina Universidad de La Sabana Chía Colombia; 2 Escuela de Salud Pública, Departamento de Epidemiología, Universidad de Michigan, Ann Arbor, Estados Unidos Escuela de Salud Pública Departamento de Epidemiología Universidad de Michigan Ann Arbor Estados Unidos; 3 Fundación para investigación en Salud y Nutrición, Bogotá, Colombia Fundación para investigación en Salud y Nutrición Bogotá Colombia

**Keywords:** micronutrientes, seguridad alimentaria y nutricional, escolares, niño, Colombia, Micronutrient, food and nutrition security, schoolchildren, child, Colombia

## Abstract

**Introducción.:**

La mitad de los hogares colombianos padecen inseguridad alimentaria. Esta se ha asociado con malnutrición, la que, según algunos estudios, podría reflejarse en un déficit de micronutrientes en los niños, aunque los datos no son concluyentes.

**Objetivo.:**

Establecer la asociación entre la inseguridad alimentaria y los niveles de hemoglobina, hierro, vitamina A, vitamina B12, folato y cinc, en escolares de Bogotá.

**Materiales y métodos.:**

Se hizo un estudio de corte transversal. Se aplicó la escala del *Household Food Security Survey Module* (HFSSM) validada en español en una muestra de hogares de escolares de Bogotá, para establecer la prevalencia de inseguridad alimentaria. Utilizando el índice de propensión, se exploró la asociación entre la inseguridad alimentaria, el hambre grave y las concentraciones de hierro, vitamina A, folato, vitamina B12 y cinc, estimadas en muestras de suero provenientes de los escolares.

**Resultados.:**

Se incluyeron 2.660 escolares. En el 76 % de los hogares había inseguridad alimentaria, de los cuales el 11,6 % se clasificaba como inseguridad alimentaria con hambre grave. La deficiencia marginal de vitamina B12 fue del 17 % y las de vitamina A y cinc, de 14 y 1,4 %, respectivamente. Aunque se encontraron niveles promedios más bajos de vitamina A (-0,009 μmol/L; IC95% -0,13 - 0,03 μmol/L), vitamina B12 (-19,57 pmol/L; IC95% -72,55 - 29,94 pmol/L) y folato (-9,25 nmol/L; IC95% -29,55 - 18,66 nmol/L) en los niños expuestos a inseguridad alimentaria con hambre grave, al compararlos con los de los no expuestos, las diferencias no fueron estadísticamente significativas.

**Conclusiones.:**

La inseguridad alimentaria con hambre grave no se asoció los valores de micronutrientes o sus deficiencias en los escolares. La escala del HFSSM mide adecuadamente la dificultad en la adquisición de alimentos por falta de recursos, pero no permite establecer una asociación con las concentraciones de micronutrientes.

## Abstract (en)

**Introduction:**

Half of the Colombian households experience some degree of food insecurity. Food insecurity has been associated with malnutrition, which could result in micronutrient deficiencies in children; however, the evidence is not conclusive.

**Objective:**

To examine the associations between food insecurity and blood concentrations of hemoglobin, ferritin, vitamin A, vitamin B12, folate, and zinc in school-age children from Bogotá, Colombia.

**Materials and methods:**

We conducted a cross-sectional study among 2,660 children aged 5-12 from Bogotá's public schools. We assessed their household food insecurity level with the Spanish version of the Household Food Security Survey Module (HFSSM), a validated scale, and quantified blood biomarkers of iron, vitamin A, folate, vitamin B12, and zinc status. We examined the associations between food insecurity, severe hunger, and micronutrient status biomarkers using propensity scores.

**Results:**

Three-quarters of households had some degree of food insecurity and 12 % had food insecurity and severe hunger. Prevalence of marginal vitamin B12 status and vitamin A and zinc deficiencies were, respectively, 17%, 14%, and 1.4%. Compared with children from households without severe hunger, those exposed to it had lower adjusted mean concentrations of vitamin A, vitamin B12, and folate, but these differences were not statistically significant.

**Conclusion:**

Food insecurity with severe hunger was not associated with micronutrient status biomarkers in Colombian school-age children. The HFSSM may adequately measure hardship in food acquisition due to lack of resources, but it does not yield an index that is associated with micronutrient status biomarkers.

Existe seguridad alimentaria "cuando todas las personas tienen en todo momento acceso físico, social y económico a suficientes alimentos inocuos y nutritivos para satisfacer sus necesidades alimenticias y sus preferencias en cuanto a los alimentos a fin de llevar una vida activa y sana" ^1^, por lo que la inseguridad alimentaria se define como la disponibilidad limitada de alimentos nutricionalmente adecuados e inocuos en formas socialmente aceptables o la capacidad incierta para adquirirlos ^2^. Esta se ve influenciada por factores de orden social y económico, como el desempleo, la inequidad social, el desplazamiento forzado, la infraestructura vial, el inadecuado uso de las tierras para fines diferentes a la agricultura y la publicidad que favorece el consumo de productos alimenticios de bajo perfil nutricional, especialmente en los países en vías de desarrollo ^3^^-^^7^. Algunos de los métodos para medir la inseguridad alimentaria son la evaluación antropométrica, las entrevistas sobre ingestión alimentaria individual (recordatorio de 24 horas y frecuencia de consumo de alimentos) y las escalas de percepción de la seguridad alimentaria en el hogar, siendo estas últimas el método más utilizado ^2^^,^^8^^,^^9^. La primera de estas escalas, la del módulo HFSSM *(Household Food Security Survey Module)* del *United States Department of Agriculture* (USDA), se originó en los años 90 en Estados Unidos; ha sido adaptada y validada en diferentes países, incluido Colombia, y ha demostrado caracterizar adecuadamente la inseguridad alimentaria en las poblaciones en las que se ha aplicado ^2^^,^^10^^-^^15^. Sin embargo, en algunos estudios realizados en el país se ha sugerido que debe tenerse cuidado al interpretar sus resultados, pues podrían dar lugar a subestimar el fenómeno cuando se compara con medidas objetivas que evalúan el consumo de energía y los nutrientes ^16^^-^^18^.

Con esta herramienta, según la Encuesta Nacional de la Situación Nutricional en Colombia del 2015, se estimó que el 54,2 % de los hogares en Colombia estaba expuesto a algún grado de inseguridad alimentaria, lo que significa que más de la mitad de la población no tenía los suficientes recursos económicos para acceder a alimentos de buena calidad nutricional (son los más costosos del mercado), lo que puede aumentar las probabilidades de que se presenten problemas de malnutrición en los hogares donde hay niños, situación que ya se ha reportado en varios estudios mediante mediciones antropométricas que han evidenciado casos de desnutrición o sobrepeso tanto en niños como en adultos en situación de inseguridad alimentaria ^3^^,^^11^^,^^19^^-^^23^.

Frecuentemente, estos estados de malnutrición, especialmente en la población infantil, se han asociado con déficit de micronutrientes, lo que influye negativamente en su crecimiento y desarrollo, ya que muchos están relacionados con funciones estructurales, neurológicas e inmunológicas y como coadyuvantes en reacciones enzimáticas del organismo. Se ha determinado que micronutrientes como el hierro, la vitamina A, la vitamina B12 y el cinc son críticos en la infancia. Su medición ayuda a comprender el fenómeno denominado "hambre oculta" (déficit de vitaminas y minerales), que es el resultado de la ingestión inadecuada de los alimentos que los proveen (carnes, lácteos, frutas y verduras). Esto no solo se ve en casos de desnutrición, sino también en individuos con exceso de peso, en quienes hay un alto consumo calórico pero una baja ingestión de micronutrientes esenciales. Las deficiencias de micronutrientes desencadenan problemas de salud como trastornos de visión o de la piel y deterioro del sistema inmunitario (déficit de vitamina A), así como anemia ferropénica, disminución del rendimiento académico y deficiencias en el aprendizaje, y una mayor propensión a infecciones (déficit de hierro), anemia megaloblástica o alteraciones neurológicas (déficit de vitamina B12), alteraciones en la síntesis de ADN y ARN (déficit de folato) o retraso en el crecimiento y la maduración sexual, y alteraciones del sistema inmunitario por deficiencia de cinc ^19^^,^^24^^-^^27^.

Aunque en Colombia se han diseñado políticas para reducir la prevalencia de las deficiencias de micronutrientes, como la fortificación de la harina de trigo con hierro y vitaminas del complejo B ^28^. Dichas deficiencias continúan siendo importantes debido a las desfavorables condiciones socioeconómicas de las familias y a la transición nutricional, lo cual, sumado a los estilos de vida que favorecen el sedentarismo, ha incrementado las prevalencias de sobrepeso y obesidad ^5^^,^^19^.

Se ha observado que las familias con inseguridad alimentaria tienen patrones de alimentación caracterizados por el bajo consumo de proteína animal ^11^^,^^29^, por lo que es probable que no se cubran en su totalidad las necesidades nutricionales de sus integrantes y ello se refleje en las concentraciones de micronutrientes en sangre. Por ello, en diversos estudios los esfuerzos se han enfocado en estudiar si la inseguridad alimentaria se asocia con los déficits de micronutrientes y en algunos se ha demostrado que tal asociación existe. Tal es el caso de dos estudios realizados en Estados Unidos con lactantes y niños de 12 a 15 años, otro en Nicaragua en niños de 3 a 11 años, y otro en México en mujeres de 20 a 49 años, en quienes se observó anemia cuando estaban expuestas a inseguridad alimentaria ^29^^-^^32^. En una revisión sistemática que analizó tres estudios en niños menores de 5 años en Etiopía, se obtuvieron resultados similares ^33^. Sin embargo, en otros estudios en niños y adolescentes menores de 19 años no se encontró esa asociación, aunque hay que tener en cuenta que en estos se analizaron otros micronutrientes ^20^^,^^32^^,^^34^^-^^36^.

Dadas las altas prevalencias de inseguridad alimentaria en Colombia y sus efectos deletéreos en el estado nutricional de la población infantil y el hecho de que los estudios previos no han sido muy concluyentes, el objetivo del presente estudio fue establecer la asociación entre la inseguridad alimentaria, evaluada mediante la escala del HFSSM, y los niveles de micronutrientes reconocidos como marcadores de malnutrición infantil, en una muestra representativa de escolares pertenecientes a los colegios públicos de Bogotá.

## Materiales y métodos

Se hizo un estudio de corte transversal con componente analítico a partir de datos provenientes de la cohorte de escolares de Bogotá conformada en febrero del 2006 mediante el reclutamiento aleatorio de 3.202 niños de 5 a 12 años que estudiaban en las escuelas públicas de Bogotá y pertenecían a los estratos socioeconómicos medio y bajo. En el inicio del reclutamiento, se pidió a los padres responder un cuestionario en el que se recolectó información sobre las características sociodemográficas y la escala de seguridad alimentaria de los hogares; también, se tomaron las mediciones antropométricas y muestras de sangre a los niños, que fueron transportadas y almacenadas en el Instituto Nacional de Salud siguiendo los protocolos previamente establecidos. Los detalles sobre los procedimientos para la toma de la muestra y los métodos utilizados para la determinación de los micronutrientes pueden encontrarse en publicaciones previas referentes a esta cohorte ^37^^,^^38^. El protocolo de investigación fue aprobado por el Comité de Ética de la Facultad de Medicina de la Universidad Nacional de Colombia, en tanto que el Comité de Ética de la Universidad de Michigan aprobó el uso de los datos del estudio ^11^^,^^37^.

### Evaluación de la inseguridad alimentaria

Se utilizó la escala del HFSSM en la versión validada en español ^39^. Esta escala se basa en la idea de que hay diferentes grados de seguridad alimentaria y una secuencia ordenada de condiciones y comportamientos, lo que resulta en patrones en los que la respuesta afirmativa a una pregunta particular tiende a condicionar a los encuestados para responder de la misma manera todas aquellas preguntas que dan una calificación en la escala; la suma de estas respuestas afirmativas permite caracterizar la inseguridad alimentaria. Según el grado de dicha inseguridad en el hogar, se puede establecer cuán afectada se ve la consecución de alimentos por falta de recursos y cómo ello genera modificaciones en el tipo, cantidad y calidad de la dieta, lo que en su fase más grave afecta no solamente a los adultos, sino también, a los niños ^10^.

La escala consta de 16 preguntas que se refieren a los 30 días previos a su aplicación y permite clasificar la inseguridad alimentaria en cuatro niveles: 1) seguridad alimentaria, es decir, hogares en que no la hay o esta es mínima (entre 0 y 2 respuestas afirmativas); 2) inseguridad alimentaria sin hambre, cuando esta es evidente y es posible observar reducción en la calidad de la dieta, acompañada o no de la reducción en la cantidad de alimentos ingeridos (entre 3 y 7 respuestas afirmativas); 3) inseguridad alimentaria con hambre moderada, cuando la ingestión de alimentos en los adultos se reduce y con frecuencia experimentan hambre debido a la falta de recursos para proveerse de alimentos, pero los niños, cuando los hay, aún no experimentan esta situación (entre 8 y 12 respuestas afirmativas), y 4) inseguridad alimentaria con hambre grave, es decir, cuando la ingestión de los niños también se ve reducida y llegan a experimentar hambre; en esta categoría, sea en hogares con o sin niños, los adultos experimentan en mayor medida la reducción en la ingestión de alimentos (con la omisión de alguna comida o varias durante el día) (entre 13 y 16 preguntas afirmativas) ^10^^,^^11^^,^^40^.

### Análisis estadístico

Se consideraron como resultados positivos, la presencia de anemia y las deficiencias de micronutrientes (hierro, vitamina A, vitamina B12, folato y cinc). La concentración de hemoglobina para establecer la anemia fue por debajo de 12,7 mg/dl para este grupo de edad, es decir, 11,5 mg/dl más 1,2 mg/dl, correspondientes al ajuste por la altura de 2.500 m ^41^. El déficit de hierro se determinó a partir de valores de ferritina menores de 15 μg/L si la proteína C reactiva tenía valores de 10 μg/L o menos. Las concentraciones de retinol menores de 0,7 μmol/L se establecieron como deficiencia de vitamina A. La deficiencia marginal de vitamina B_12_ correspondió a valores por debajo de 222 μmol/L y, como deficiencia de folato y cinc, los valores de referencia fueron menos de 305 nmol/L y menos de 65 μg/dl, respectivamente ^3^^,^^36^^,^^42^.

Después de explorar varios puntos de corte, se encontró que el que separaba la inseguridad alimentaria con hambre grave de los demás grados, correspondía al que mejor discriminaba esta exposición, por lo cual, de acuerdo con el puntaje obtenido en la escala HFSSM, se definió que el grupo no expuesto sería aquel que agrupaba las categorías de seguridad alimentaria, inseguridad alimentaria sin hambre e inseguridad alimentaria con hambre moderada, mientras que el grupo expuesto sería la categoría de inseguridad alimentaria con hambre grave, pues es en este nivel en donde los niños se ven más afectados y experimentan una reducción de la ingestión y de la sensación de hambre.

Se recolectó la información sociodemográfica y antropométrica de todos los niños: edad y educación de la madre, condición de madre soltera, si la casa era propia y el estrato socioeconómico; edad del niño, sexo, indicador de la talla para la edad e índice de masa corporal (IMC). Estos últimos incluían como variables el cálculo de los puntajes Z del IMC y la talla para la edad, según la referencia para niños y adolescentes de la Organización Mundial de la Salud (OMS) ^43^. Según el IMC para la edad, el bajo peso correspondió a un puntaje Z por debajo de -1, el peso adecuado, a uno entre ≥ a -1 y ≤ a 1, el sobrepeso, a uno entre >1 a ≤ 2, y la obesidad, a uno > de 2; el retraso en el crecimiento se definió como un puntaje z menor de -2 ^44^^,^^45^.

**Figura 1:**
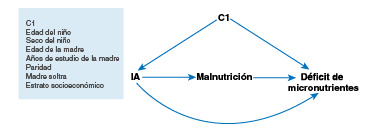
Gráfico acíclico dirigido *(Directed Acyclic Graph,* DAG) de la asociación entre inseguridad alimentaria y déficit de micronutrientes

En la figura 1 se representa la relación de las variables sociodemográficas y antropométricas con la inseguridad alimentaria, el de déficit de micronutrientes y el probable efecto de confusión que ejercen en su asociación. Para comparar el grupo de expuestos y el de no expuestos con el déficit de micronutrientes, y dado el posible desequilibrio en las características básales de cada grupo, estas se equilibraron mediante el índice de propensión ^46^. Para controlar las variables de confusión, se determinó el índice de propensión de la probabilidad de estar expuesto a la inseguridad alimentaria con hambre grave a partir de las características sociodemográficas y antropométricas, con el fin de obtener el equilibrio de las características básales del grupo no expuesto (seguridad alimentaria, inseguridad alimentaria sin hambre y con hambre moderada) y del expuesto (inseguridad alimentaria con hambre grave).

**Figura 2:**
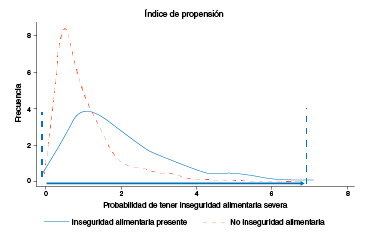
Zona de soporte común para garantizar probabilidades similares en el grupo de los expuestos (inseguridad alimentaria con hambre grave) y el de los no expuestos (seguridad alimentaria, inseguridad alimentaria sin hambre e inseguridad alimentaria con hambre moderada)

**Figura 3:**
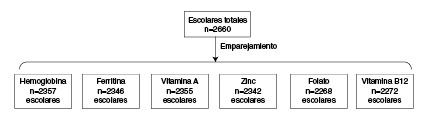
Reducción del número de la muestra después de emparejar

A continuación, se determinó la zona de soporte común para establecer el rango de valores del índice de propensión en el cual coincidían los niños expuestos a la inseguridad alimentaria con hambre grave y los no expuestos (figura 2), con el fin de disminuir las diferencias de las características básales entre los dos grupos. En el paso siguiente, se hizo el emparejamiento utilizando el método de distancia máxima de calibración (0,005) para asignar los individuos no expuestos a cada uno de los individuos expuestos. Según el biomarcador analizado, el número total de niños seleccionados estuvo entre 2.268 y 2.357 de la muestra inicial de 2.660 niños (figura 3). A continuación, se evaluó el adecuado equilibrio entre las características de los dos grupos a partir de las diferencias estandarizadas y el índice de Rubin ^47^^,^^48^. Por último, se calculó el efecto del tratamiento en los tratados *(Average Treatment effect on the Treated,* ATT) mediante la prueba t de Student para variables continuas con un intervalo de confianza del 95 % y, así, determinar la diferencia de los niveles de micronutrientes entre los expuestos a inseguridad alimentaria con hambre grave y los no expuestos. Todos los análisis se hicieron con el programa estadístico Stata 14.

## Resultados

El promedio de edad de los niños fue de 8,7 (±1,8) años, el 49,6 % eran niñas, alrededor de la cuarta parte eran madres solteras y la mayoría no tenía casa propia (cuadro 1). En cuanto al estado nutricional de los niños, se encontró una mayor prevalência de exceso de peso (cerca de 19 %) comparado con el bajo peso (12,5 %). Por otra parte, el retraso en la talla se presentó en el 9,7 % de la población. Como se observa en el cuadro 2, la deficiencia marginal de vitamina B12 fue la más prevalente en esta población (17 %), la deficiencia de vitamina A se encontró en un 14 %, en tanto que la de micronutrientes como el cinc y el folato registraron las menores prevalencias; de este último solo se registraron dos casos de niños con concentraciones menores de 305 nmol/L.

**Cuadro 1:** Características generales de los escolares participantes en el estudio en el 2006 según grupo de expuestos y no expuestos

Característica		Total	No expuestos	Expuestos	^**^ **p**
Edad del niño, años	Media (ds)	8,7 (1,8)	8,7 (1,8)	9,0 (1,9)	0,99
Sexo, niña	% (n)	49,6 (1319)	49,2 (1157)	52,4 (162)	0,28
Retardo en talla, Talla/edad	% (n)	9,7 (250)	9,1 (207)	14,1 (43)	0,01
IMC niño, IMC/edad					0,04
Bajo peso	% (n)	12,4 (321)	12,0 (274)	15,5 (47)	
Adecuado	% (n)	68,9 (1778)	68,9 (1568)	69,3 (210)	
Sobrepeso	% (n)	14,5 (373)	14,9 (338)	11,6 (35)	
Obesidad	% (n)	4,1 (107)	4,2 (96)	3,6 (11)	
Hemoglobina g/dl	Media (ds)	14,5 (1,2)	14,5 (1,2)	14,5 (1,1)	0,32
Ferritina μg/L	Media (ds)	42,2 (23,1)	42,2 (23,0)	42,1 (24,1)	0,49
Vitamina A μmol/L	Media (ds)	1,04 (0,35)	1,04 (0,35)	1,02 (0,33)	0,22
Cinc μ/dl	Media (ds)	41,6 (140,5)	41,5 (140,4)	42,3 (141,0)	0,58
Folato nmol/L	Media (ds)	858,5 (258,5)	860 (262,3)	847,2 (228,9)	0,21
Vitamina B12 μmol/L	Media (ds)	327,6 (106,4)	329,9 (107,1)	310,5 (99,8)	<0,01
Proteína C reactiva mg/L	Media (ds)	1,5 (2,6)	1,5 (2,7)	1,3 (1,9)	0,08
Edad de la madre, años	Media (ds)	26,6 (6,5)	26,4 (6,4)	28,1 (6,7)	1,00
Años de estudio madre	Media (ds)	8,7 (3,3)	8,9 (3,3)	7,1 (3,4)	<0,01
Número de hijos					<0,01
1	% (n)	11,6 (290)	12,6 (277)	4,4 (13)	
2	% (n)	35,7 (893)	37,5 (827)	22,5 (66)	-
3	% (n)	30,3 (758)	30,3 (668)	30,9 (90)	
4	% (n)	12,7 (317)	11,6 (255)	21,2 (62)	
5	% (n)	9,6 (240)	8,1 (178)	21,2 (62)	-
Madre soltera	% (n)	27,1 (675)	25,5 (561)	39,2 (114)	<0,01
Casa propia, no	% (n)	66,6 (1762)	65,2 (1524)	77,5 (238)	<0,01
Estrato socioeconómico	% (n)				<0,01
1	% (n)	7,0 (186)	6,6 (154)	10,4 (32)	
2	% (n)	32,3 (858)	31,6 (741)	37,9 (117)	-
3	% (n)	53 (1407)	53,8 (1261)	47,3 (146)	
4	% (n)	7,6 (202)	8,0 (188)	4,5 (14)	-
Inseguridad alimentaria (IA)					
Sin IA % (n)		24 (640)	-	-	
IA sin hambre % (n)		46,5 (1237)			
IA con hambre moderada % (n)		17,8 (474)	-	-	
IA con hambre grave % (n)		11,6 (309)	-	-	

^**^ Análisis sin ajustar

En el 75,9 % de las familias, se registraba algún nivel de inseguridad alimentaria: la más prevalente fue aquella sin hambre, con un 46,5 %, en tanto que con hambre moderada y grave se registró en el 17,8 y el 11,6 %, respectivamente.

Una vez dicotomizada la variable de exposición, se hicieron análisis bivariados sin ajustar y se encontró que el retraso en el crecimiento y el bajo peso se asociaban a la inseguridad alimentaria con hambre grave, así como a las variables sociodemográficas de años de estudio de la madre, número de hijos, si se trataba de madres solteras, si la casa no era propia, y estrato socioeconómico, con valores de p<0,05 (cuadro 1). Para el caso de los micronutrientes, no se encontró asociación entre su deficiencia y la inseguridad alimentaria con hambre grave, pero sí se observaron menores niveles de vitamina B12 en los niños expuestos a esta con hambre grave comparados con los no expuestos (310,5 *Vs.* 329,9 pmol/L; p<0,01), en tanto que los niveles de los otros biomarcadores registraron valores de p>0,05 (cuadros 1 y 2).

**Cuadro 2:** Prevalencia de anemia y déficit de micronutrientes en los escolares participantes en el estudio en el 2006

Característica	Total	No expuestos	Expuestos	^**^ **p**
Anemia (Hb <12,7 g/dl ^*^)				0,95
Sí % (n)	3,5 (93)	3,5 (82)	3,6 (11)	
No % (n)	96,5 (2564)	96,5 (2266)	96,4 (298)	
Deficiencia de hierro				0,85
Sí % (n)	3,2 (82)	3,2 (73)	3,0 (9)	
No % (n)	96,8 (2514)	96,8 (2223)	97,0 (291)	
Deficiencia de vitamina A				0,93
Sí % (n)	13,9 (369)	13,9 (326)	14,1 (43)	
No % (n)	86,1 (2287)	86,1 (2024)	86 (263)	
Deficiencia de cinc				0,84
Sí % (n)	1,4 (38)	1,5 (34)	1,3 (4)	
No % (n)	98,6 (2599)	98,5 (2298)	98,7 (301)	
Deficiencia de folato				-
No % (n)	100 (2560)	100 (2257)	100 (303)	
Deficiencia marginal de vitamina B12				0,18
Sí % (n)	16,7 (427)	16,3 (369)	16,7 (58)	
No % (n)	83,4 (2138)	83,7 (1896)	80,7n (242)	

^*^ Punto de corte ajustado por altura ^41^

^**^ Análisis sin ajustar

Utilizando el índice de propensión, se estimaron las probabilidades de estar expuesto a inseguridad alimentaria con hambre grave y de no estar expuesto, y se determinó la zona de soporte común que, como se puede observar en la figura 2, es amplia e incluyó a la mayoría de los niños, lo que indica que tanto el grupo expuesto como el no expuesto tenían características basales parecidas.

Como se observa en los cuadros 3 y 4, antes del emparejamiento todas las características basales de la población, excepto el sexo, se encontraban desequilibradas, pero una vez se emparejaron, se garantizó el adecuado equilibrio de las variables de confusión en el grupo expuesto y en el no expuesto para cada uno de los biomarcadores. Esto resultó en diferencias de medias estandarizadas de <10 % y valores del índice de Rubin que pasaron de 91 a <7 para cada biomarcador.

**Cuadro 3:** Variables seleccionadas antes y después del emparejamiento para hemoglobina, ferritina y vitamina A en los escolares participantes en el estudio en el 2006

Variable	Hemoglobina						Ferritina						Vitamina A					
	Antes del emparejamiento			Después del emparejamiento			Antes del emparejamiento			Después del emparejamiento			Antes del emparejamiento			Después del emparejamiento		
	IA + hambre grave	No expuesto	^*^ **% DE**	IA + hambre grave	No expuesto	^*^ **%** **DE**	IA + hambre grave	No expuesto	^*^ **%** **DE**	IA + hambre grave	No expuesto	^*^ **%** **DE**	IA + hambre grave	No expuesto	^*^ **%** **DE**	IA + hambre grave	No expuesto	^*^ **%** **DE**
Edad de la madre	28,2	26,4	27,4	28,1	28,3	-2,8	28,2	26,4	27,3	28,2	28,2	-0,6	28,5	26,4	27,8	28,1	28,1	-0,3
Madre soltera	0,4	0,3	30,2	0,4	0,4	1,1	0,4	0,2	30,8	0,4	0,4	0,5	0,4	0,3	30,3	0,4	0,4	0,6
Años de estudio																		
de la madre	7,1	8,9	-54,1	7,2	7,2	1,3	7,1	8,9	-53,1	7,2	7,2	2	7,1	8,9	-53,0	7,3	7,3	-0,6
Número de hijos	3,3	2,6	60,5	3,3	3,2	2,6	3,3	2,6	59,7	3,3	3,2	2,4	3,3	2,6	61,2	3,3	3,3	1,4
Casa propia	0,2	0,3	-24,8	0,2	0,2	2	0,2	0,3	-24,5	0,2	0,2	-0,3	0,2	0,3	-24,3	0,2	0,2	1,8
Estrato																		
socioeconómico	2,4	2,6	-26,1	2,5	2,5	-0,5	2,4	2,6	-25,8	2,5	2,4	2,7	2,4	2,6	-25,9	2,5	2,5	0,1
Sexo del niño	0,5	0,5	-7,7	0,5	0,5	-2,2	0,5	0,5	-8,8	0,5	0,5	-2,8	0,5	0,5	-8,2	0,5	0,5	-2,6
Edad del niño	9,0	8,6	21,3	9,0	9,0	-2,2	9,0	8,6	21,4	9,0	9,0	-0,2	9,0	8,6	21,8	9,0	9,1	-4,0
IMC niño	-0,06	0,17	-22,9	-0,05	-0,06	0,6	-0,07	0,17	-23,3	-0,06	-0,07	1	-0,07	0,17	-23,7	-0,06	-0,07	1,40
Índice de Rubin	91,7			6,5			91,2			5,8			91,5			5,9		

IA: inseguridad alimentaria

*% DE: porcentaje de diferencias estandarizadas

**Cuadro 4:** Variables seleccionadas antes y después del emparejamiento para cinc, folato y vitamina B12 en los escolares participantes en el estudio en el 2006

Variable	Cinc						Folato						Vitamina B12					
	Antes del emparejamiento			Después del emparejamiento			Antes del emparejamiento			Después del emparejamiento			Antes del emparejamiento			Después del emparejamiento		
	IA + hambre grave	No expuesto	^*^ **% DE**	IA + hambre grave	No expuesto	^*^ **%** **DE**	IA + hambre grave	No expuesto	^*^ **%** **DE**	IA + hambre grave	No expuesto	^*^ **%** **DE**	IA + hambre grave	No expuesto	^*^ **%** **DE**	IA + hambre grave	No expuesto	*% DE
Edad de la madre	28,2	26,4	27,4	28,2	28,1	0,9	28,3	26,4	28,1	28,2	28,0	2,0	28,3	26,4	28,7	28,2	28,1	1,9
Madre soltera	0,4	0,2	30,7	0,4	0,4	0,3	0,4	0,2	31,6	0,4	0,4	-0,2	0,4	0,2	29,9	0,4	0,4	1,3
Años de estudio,																		
madre	7,1	8,9	-53,8	7,2	7,2	-0,4	7,1	8,9	-53,1	7,2	7,2	1,4	7,1	8,9	-51,6	7,2	7,2	2,1
Número de hijos	3,3	2,6	60,4	3,3	3,3	0,8	3,3	2,6	59,2	3,3	3,2	1,5	3,3	2,6	60,6	3,3	3,3	0,4
Casa propia	0,2	0,3	-24,5	0,2	0,2	1,5	0,2	0,3	-22,4	0,2	0,2	-0,3	0,2	0,3	-24,0	0,2	0,2	-0,8
Estrato																		
socioeconómico	2,4	2,6	-25,6	2,5	2,5	0	2,4	2,6	-26,4	2,4	2,4	2,0	2,5	2,6	-24,4	2,5	2,5	0,4
Sexo del niño	0,5	0,5	-8,2	0,5	0,5	-2,2	0,5	0,5	-8,2	0,5	0,5	-2,4	0,5	0,5	-7,5	0,5	0,5	-1,6
Edad del niño	9,0	8,6	22,3	9,0	9,1	-2	9,0	8,7	20,6	9,0	9,0	-0,8	9,1	8,7	21,9	9,1	9,1	-3,1
IMC del niño	-0,07	0,17	-23,4	-0,06	-0,08	1,3	-0,06	0,17	-22,5	-0,05	-0,06	0,8	-0,06	0,17	-22,4	-0,05	-0,06	0,8
Índice de Rubin	91,6			3,8			90,5			4,5			90,0			4,7		

IA: inseguridad alimentaria

*% DE: porcentaje de diferencias estandarizadas

Al estimar el ATT, se encontró que los niños expuestos a inseguridad alimentaria con hambre grave tenían menores niveles de hemoglobina, vitamina A, folato y vitamina B12 que los no expuestos; sin embargo, estas diferencias no fueron estadísticamente significativas al contrastar con los valores de p>0,05 y con los intervalos de confianza, los cuales denotaban una gran imprecisión al ser muy amplios e incluir el cero, lo que anula la asociación (cuadro 5).

**Cuadro 5:** Efecto del tratamiento en los tratados (ATT) para cada biomarcador en los escolares participantes en el estudio en el 2006

Biomarcador	Media expuestos a IA + hambre grave	Media no expuestos	Diferencia	p	IC_95%_
Hemoglobina	14,51	14,55	-0,04	0,36	-0,29 - 0,11
Ferritina	42,56	41,67	0,89	0,76	-5,80 - 4,28
Vitamina A	1,011	1,020	-0,009	0,27	-0,13 - 0,03
Cinc	141,69	139,31	2,38	0,71	-7,74 - 11,33
Folato	839,02	858,60	-19,57	0,29	-72,55 - 29,94
Vitamina B12	311,22	320,47	-9,25	0,65	-29,55 - 18,66

## Discusión

En este estudio se exploró la asociación entre la inseguridad alimentaria y los niveles de micronutrientes en niños escolares de estrato socioeconómico medio y bajo; se observaron niveles un poco menores de vitamina A, folato y vitamina B12 en los niños con inseguridad alimentaria y hambre grave, comparados con quienes no estaban expuestos a esta o lo estaban, pero sin hambre o con hambre moderada. Sin embargo, esas diferencias no fueron estadísticamente significativas, por lo que no se logró establecer una asociación entre la inseguridad alimentaria con hambre grave y las concentraciones de los biomarcadores estudiados ni su deficiencia. Estos resultados concuerdan con lo encontrado en Brasil por Figueroa, en un estudio de corte transversal en niños preescolares en condiciones socioeconómicas vulnerables asistentes a jardines infantiles públicos, en quienes se establecieron los niveles de inseguridad alimentaria y los niveles séricos de retinol, hemoglobina y cinc mediante la Escala Brasileña de Inseguridad Alimentaria (EBIA); sus hallazgos no demostraron la asociación con ninguno de los biomarcadores a pesar de la alta prevalencia de inseguridad alimentaria en esa población (64,2 %) ^35^.

Resultados similares se presentaron en un estudio realizado en México con los datos sobre mujeres y adolescentes en edad fértil (15 a 49 años) recogidos en la Encuesta de Salud y Nutrición de ese país en el 2012. El objetivo era determinar la asociación entre la inseguridad alimentaria y la concurrencia de sobrepeso, obesidad y anemia. En las adolescentes no se encontró dicha asociación, pero en las mujeres adultas sí fue positiva en los casos de inseguridad alimentaria leve o moderada, lo que concurrió con la asociación entre la alta probabilidad *(odd)* de anemia en este grupo y los ítems de la escala de seguridad alimentaria usada (ELCSA) relacionados con la calidad deficiente de la dieta. En cuanto a los resultados del grupo de las adolescentes, debe señalarse que en algunos estudios se ha comprobado que los padres tienden a proteger a los niños de los efectos de la inseguridad alimentaria disminuyendo su propio consumo de alimentos en favor de ellos ^32^.

En otro estudio en niños de 6 a 14 años de escuelas de bajos recursos de dos condados de China, se evaluó la asociación entre la inseguridad alimentaria y cuatro signos de malnutrición (anemia, retraso en el crecimiento, emaciación y raquitismo) y, aunque se encontró asociación *(odds ratio,* OR, de 3,08), cuando los cuatro signos se agruparon en una sola variable denominada malnutrición grave y se incluyeron en un modelo, esta asociación no se evidenció al realizar el análisis ajustado por cada signo de manera individual ^20^. Por último, en estudios previos basados en datos de la misma cohorte estudiada aquí analizados con un modelo de ecuaciones estructurales generalizadas (GEE), estas diferencias no fueron estadísticamente significativas cuando se ajustó por las variables de confusión, aunque en los análisis univariados se reportaron niveles más bajos de vitamina B_1_2 en los niños expuestos a inseguridad alimentaria ^36^.

En contraste, aunque en nuestro estudio se reportaron mayores prevalencias de anemia en el grupo de escolares en condiciones de inseguridad alimentaria, pero sin asociación, en otro realizado en Estados Unidos a partir de los datos sobre niños y adolescentes (3-19 años) de la Encuesta Nacional de Salud y Nutrición (NHANES) del período 1999-2004, sí se encontró asociación entre la inseguridad alimentaria y la presencia de anemia en el grupo de edad de 12 a 15 años (OR=2,95). No Sucedió lo mismo en el grupo de 6 a 11 años, pues, a pesar de registrar un OR de 8, el intervalo de confianza fue muy amplio (IC_95%_ 2,07-31,36) y el número de casos era escaso, lo que explicaría que el resultado en el grupo de adolescentes pudo deberse a la gran demanda de hierro propia del crecimiento, especialmente en las niñas por la menarquia, así como a la influencia de factores ambientales y psicosociales ^29^.

Según los datos reportados en la Encuesta Nacional de la Situación Nutricional, ENSIN 2015, la población aquí analizada registró prevalencias de inseguridad alimentaria 22 puntos porcentuales por encima de las cifras nacionales (76 *Vs.* 54,2 %), en tanto que el déficit marginal de vitamina B12 fue de más del doble (16,7 *Vs.* 6,8 %). La prevalencia de deficiencia de vitamina A, en cambio, fue la mitad de la registrada para el país (13,9 *Vs.* 27,3%) ^19^. En este sentido, aunque la reducción en la prevalencia de anemia y el déficit de hierro y folato frente a lo encontrado a nivel nacional se pueda explicar por la política de fortificación de la harina de trigo establecida en 1996 y la implementación de programas de refrigerios escolares desde el 2004, que también proveen hierro adicional ^36^^,^^49^, debe señalarse que tales deficiencias en la población infantil continúan siendo importantes y propician la aparición de condiciones médicas que afectan el crecimiento y el desarrollo adecuado de los niños (problemas de visión o en la piel, anemia ferropénica o megaloblástica, retraso en el crecimiento y alteraciones inmunológicas, neurológicas y en la síntesis de ADN) ^24^.

Se podía esperar que las altas prevalencias de inseguridad alimentaria estimadas con la escala aquí empleada se reflejarían en porcentajes más altos de desnutrición en esta población, pero, por el contrario, se encontró un porcentaje mayor de niños con exceso de peso, y de peso adecuado en la gran mayoría, lo que se explicaría por la transición nutricional caracterizada por el consumo de alimentos de baja calidad nutricional que favorece la aparición de malnutrición y el consecuente aumento del componente adiposo en la composición corporal del individuo ^50^. Por ello la interpretación de los resultados de la escala debe ser cuidadosa, en especial, al establecer políticas públicas encaminadas a mitigar esta problemática, pues no permite medir este tipo de aspectos específicos ^17^^,^^18^.

Entre las limitaciones del estudio, cabe mencionar que los resultados solo corresponden a la población escolar de estratos socioeconómicos medio y bajo, por lo que no puede generalizarse a otros estratos ni grupos etarios eventualmente expuestos a algún grado de inseguridad alimentaria. Asimismo, se trató de un estudio de corte transversal en el que no fue posible determinar la causalidad entre dicha inseguridad y los niveles de micronutrientes, además de que el tiempo de evaluación de la inseguridad alimentaria (30 días) no fue suficiente para detectar las deficiencias de micronutrientes. En el caso de la vitamina A, por ejemplo, es importante considerar los hábitos alimentarios que afectan sus niveles porque esta población no acostumbra a consumir los alimentos que proveen este micronutriente.

El estudio también tiene algunas fortalezas: la información analizada provino de una cohorte establecida mediante un método de muestreo que garantizó la representatividad de la población específica de escolares de ingresos bajos y medios, aunque los datos eran del 2006. Cabe anotar que, aunque hay estudios similares, en su gran mayoría su análisis se restringe a la asociación con la anemia, pero no al déficit de otros micronutrientes como sí lo hace el nuestro, lo que le añade valor. Además, este es el primer estudio en nuestro medio que utilizó la metodología del índice de propensión, lo que permitió ajustar los análisis a partir de características basales de la población que pueden ser factores de confusión con respecto al efecto real de la inseguridad alimentaria con hambre grave en las concentraciones de los micronutrientes. Al equilibrar estos componentes en los dos grupos, se pudo determinar el efecto real, que, aunque no fue significativo, sí estableció diferencias en los niveles de algunos micronutrientes de los niños expuestos a inseguridad alimentaria con hambre grave frente a los no expuestos. Asimismo, debe señalarse que las variables equilibradas correspondían a aquellas que en otros estudios se han reportado como asociadas a la inseguridad alimentaria ^3^^,^^11^^,^^21^^,^^22^.

En conclusión, no se encontró una asociación entre la inseguridad alimentaria con hambre grave y la concentración de micronutrientes en la población escolar bajo estudio y, aunque sí se encontraron menores niveles de vitamina A, vitamina B12 y folato en los expuestos, dichas diferencias no fueron significativas. A pesar de que la escala de seguridad alimentaria mide adecuadamente la dificultad en la adquisición de alimentos por falta de recursos económicos, no necesariamente permite establecer una asociación con las concentraciones de micronutrientes, por lo que el método más preciso para su estimación es la medición de los niveles en sangre. Debe añadirse que la exposición a la inseguridad alimentaria por sí sola no es un factor que se asocie con las deficiencias de micronutrientes, pero probablemente la presencia de otros factores afecte sus niveles sanguíneos (enfermedades infecciosas, baja ingestión de los alimentos que los proveen y malabsorción debida el bajo consumo de otros nutrientes o micronutrientes necesarios para un adecuado metabolismo). Dichos factores ameritan ser explorados en futuros estudios.

## References

[1] Organización de las Naciones Unidas para la Alimentación y la Agricultura (2019). El estado de la seguridad alimentaria y la nutrición en el mundo 2019. Protegerse frente a la desaceleración y el debilitamiento de la economía.

[2] Organización de las Naciones Unidas para la Alimentación y la Agricultura (2012). Escala latinoamericana y caribeña de seguridad alimentaria (ELCSA): manual de uso y aplicaciones.

[3] Ministerio de Proteccion Social, Instituco Colombiano de Bienestar Familiar, Instituto Nacional de Salud, Profamilia (2011). Encuesta Nacional de la Situación Nutricional en Colombia 2010.

[4] Del Castillo S, Fonseca Z, Mantilla M, Mendieta N (2012). Estudio para la medición de seguridad alimentaria y nutricional en el Magdalena medio colombiano. Caso Cesar. Revista de la Facultad de Medicina.

[5] Álvarez-Castaño LS, Pérez-Isaza EJ (2013). Situación alimentaria y nutricional en Colombia desde la perspectiva de los determinantes sociales de la salud. Perspectivas en Nutrición Humana.

[6] Matamoros SE, Cortés MH, Motato ÁM (2007). Caracterización de la situación de seguridad alimentaria de la población en condición de desplazamiento forzado: una mirada a través de las familias. Estudio de caso Bogotá. Revista de la Facultad de Medicina.

[7] Royo-Bordonada MÁ (2013). ¿Pueden contribuir las industrias alimentaria y de la publicidad a prevenir la obesidad infantil y promover hábitos saludables?. Gac Sanit.

[8] Pérez-Escamilla R, Segall-Corrêa AM (2008). Food insecurity measurement and indicators. Revista de Nutrição.

[9] Salvador-Castell G, Ngo de la Cruz J, Pérez-Rodrigo C, Aranceta J (2015). Escalas de evaluación de la inseguridad alimentaria en el hogar. Revista Española de Nutrición Comunitaria.

[10] Bickel GW, Hamilton WL, Cook JT, Thompson WW, Buron LF, Frongillo EA (1997). Household food security in the United States in 1995: Summary Report of the Food Security Measurement Project.

[11] Isanaka S, Mora-Plazas M, López-Arana S, Baylin A, Villamor E (2007). Food insecurity is highly prevalent and predicts underweight but not overweight in adults and school children from Bogotá, Colombia. J Nutr.

[12] Villagómez-Ornelas P, Hernández-López P, Carrasco-Enríquez B, Barrios-Sánchez K, Pérez-Escamilla R, Melgar-Quiñónez H (2014). alidez estadística de la Escala Mexicana de Seguridad Alimentaria y la Escala Latinoamericana y Caribeña de Seguridad Alimentaria. Salud Pública México.

[13] Melgar-Quiñónez H (2010). Validación de la escala latinoamericana y caribeña para la medición de la seguridad alimentaria (ELCSA) en Guatemala.

[14] Dellohain PL, Sanjur D (2000). La adaptación y validación de una escala de seguridad alimentaria en una comunidad de Caracas, Venezuela. Arch Latinoam Nutr.

[15] Álvarez MC, Estrada A, Montoya EC, Melgar-Quiñónez H (2006). Validation of a household food security scale in Antioquia, Colombia. Salud Pública México.

[16] Jiménez S, Prada G, Herrán ÓF (2012). Escalas para medir la seguridad alimentaria en Colombia: ¿son válidas?. Rev Chil Nutr.

[17] Camargo G MI, Quintero L DC, Herrán F OF (2012). Seguridad alimentaria en Colombia y modelo Rasch. Rev Chil Nutr.

[18] Del Castillo SE, Patiño GA, Herrán ÓF (2012). Inseguridad alimentaria: variables asociadas y elementos para la política social. Biomédica.

[19] Ministerio de Salud y Protección Social, Instituto Colombiano de Bienestar Familiar, Instituto Nacional de Salud, Universidad Nacional de Colombia (2019). Encuesta Nacional de la Situación Nutricional en Colombia 2015 ENSIN.

[20] Shen X, Gao X, Tang W, Mao X, Huang J, Cai W (2015). Food insecurity and malnutrition in Chinese elementary school students. Br J Nutr.

[21] Shahraki SH, Amirkhizi F, Amirkhizi B, Hamedi S (2016). Household food insecurity is associated with nutritional status among Iranian children. Ecol Food Nutr.

[22] Abdurahman AA, Mirzaei K, Dorosty AR, Rahimiforoushani A, Kedir H (2016). Household food insecurity may predict underweightand wasting among children aged 24-59 months. Ecol Food Nutr.

[23] Schlüssel MM, Silva AAM da, Pérez-Escamilla R, Kac G (2013). Household food insecurity and excess weight/obesity among Brazilian women and children: A life-course approach. Cad Saúde Pública.

[24] Mahan LK, Escott-Stump S (2009). Krouse Dietoterapia.

[25] Muthayya S, Rah JH, Sugimoto JD, Roos FF, Kraemer K, Black RE (2013). The global hidden hunger indices and maps: An advocacy tool for action. PloS ONE.

[26] Kennedy G, Natel G, Shetty P (2003). The scourge of "hidden hunger': Global dimensions of micronutrient deficiencies.

[27] Thornton KA, Mora-Plazas M, Marín C, Villamor E (2014). Vitamin A deficiency is associated with gastrointestinal and respiratory morbidity in school-age children. J Nutr.

[28] Ministerio de Salud Pública Decreto número 1944 de 28 de octubre de 1996 por el cual se reglamenta la fortificación de la harina de trigo y se establecen las condiciones de comercialización, rotulado, vigilancia y control. 1996.

[29] Eicher-Miller HA, Mason AC, Weaver CM, McCabe GP, Boushey CJ (2009). Food insecurity is associated with iron deficiency anemia in US adolescents. Am J Clin Nutr.

[30] Park K, Kersey M, Geppert J, Story M, Cutts D, Himes JH (2009). Household food insecurity is a risk factor for iron-deficiency anaemia in a multi-ethnic, low-income sample of infants and toddlers. Public Health Nutr.

[31] Schmeer KK, Piperata BA (2017). Household food insecurity and child health. Matern Child Nutr.

[32] Jones AD, Mundo-Rosas V, Cantoral A, Levy TS (2017). Household food insecurity in Mexico is associated with the co-occurrence of overweight and anemia among women of reproductive age, but not female adolescents. Matern Child Nutr.

[33] Belachew A, Tewabe T (2020). Under-five anemia and its associated factors with dietary diversity, food security, stunted, and deworming in Ethiopia: Systematic review and meta-analysis. Syst Rev.

[34] Pirkle CM, Lucas M, Dallaire R, Ayotte P, Jacobson JL, Jacobson SW (2014). Food insecurity and nutritional biomarkers in relation to stature in Inuit children from Nunavik. Can J Public Health.

[35] Pedraza DF, Queiroz D de, Paiva A de A, Cunha MAL da, Lima ZN (2014). Food security, growth and vitamin A, hemoglobin and zinc levels of preschool children in the northeast of Brazil. Cien Saude Colet.

[36] Villamor E, Mora-Plazas M, Forero Y, López-Arana S, Baylin A (2008). Vitamin B-12 status is associated with socioeconomic level and adherence to an animal food dietary pattern in Colombian school children. J Nutr.

[37] Arsenault JE, Mora-Plazas M, Forero Y, López-Arana S, Marín C, Baylin A (2009). Provision of a school snack is associated with vitamin B-12 status, linear growth, and morbidity in children from Bogotá, Colombia. J Nutr.

[38] Maslova E, Mora-Plazas M, Forero Y, López-Arana S, Baylin A, Villamor E (2009). Are vitamin A and iron deficiencies re-emerging in urban Latin America? A survey of schoolchildren in Bogotá, Colombia. Food Nutr Bull.

[39] Harrison GG, Stormer A, Herman DR, Winham DM (2003). Development of a Spanish-language version of the U.S. household food security survey module. J Nutr.

[40] Bickel G, Mark N, Price C, Hamilton W, Cook J (2000). Guide to Measuring Household Food Security, Revised 2000. U.S.

[41] World Health Organization - WHO (2011). Haemoglobin concentrations for the diagnosis of anaemia and assessment of severity. Vitamin and Mineral Nutrition Information System.

[42] Robinson SL, Marín C, Oliveros H, Mora-Plazas M, Richards BJ, Lozoff B (2018). Iron deficiency, anemia, and low vitamin B-12 serostatus in middle childhood are associated with behavior problems in adolescent boys: Results from the Bogotá school children cohort. J Nutr.

[43] de Onis M, Onyango AW, Borghi E, Siyam A, Nishida C, Siekmann J (2007). Development of a WHO growth reference for school-aged children and adolescents. Bull World Health Organ.

[44] Organización Panamericana de la Salud, Organización Mundial de la Salud (2008). Curso de capacitación sobre la evaluación del crecimiento del niño.

[45] Ministerio de Salud y Protección Social Resolución 2465 de 2016, Por la cual se adoptan los indicadores antropométricos, patrones de referencia y puntos de corte para la clasificación antropométrica del estado nutricional de niñas, niños y adolescentes menores de 18 años de edad, adultos de 18 a 64 años de edad y gestantes adultas y se dictan otras disposiciones.

[46] Johnson SR, Tomlinson GA, Hawker GA, Granton JT, Feldman BM (2018). Propensity score methods for bias reduction in observational studies of treatment effect. Rheum Dis Clin North Am.

[47] Vélez M, Egurrola J, Jaimes Barragán F (2013). Ronda clínica y epidemiológica. Uso de la puntuación de propensión (propensity score) en estudios no experimentales. Iatreia.

[48] Shenyang G, Mark WF (2015). Statistical Methods and Applications.

[49] Perng W, Mora-Plazas M, Marin C, Villamor E (2013). Iron status and linear growth: A prospective study in school-age children. Eur J Clin Nutr.

[50] Shroff MR, Perng W, Baylin A, Mora-Plazas M, Marin C, Villamor E (2014). Adherence to a snacking dietary pattern and soda intake are related to the development of adiposity: A prospective study in school-age children. Public Health Nutr.

